# The role of stress echocardiography in transcatheter aortic valve implantation and transcatheter edge-to-edge repair era: A systematic review

**DOI:** 10.3389/fcvm.2022.964669

**Published:** 2022-11-16

**Authors:** Rita Pavasini, Gioele Fabbri, Nicola Bianchi, Maria Angela Deserio, Federico Sanguettoli, Luca Zanarelli, Elisabetta Tonet, Giulia Passarini, Matteo Serenelli, Gianluca Campo

**Affiliations:** Cardiology Unit, Azienda Ospedaliero-Universitaria di Ferrara, Ferrara, Italy

**Keywords:** stress echocardiography, Mitraclip, TAVI, aortic stenosis, mitral regurgitation, TEER

## Abstract

**Objectives:**

In the last decade, percutaneous treatment of valve disease has changed the approach toward the treatment of aortic stenosis (AS) and mitral regurgitation (MR). The clinical usefulness of stress echocardiography (SE) in the candidates for transcatheter aortic valve implantation (TAVI) and transcatheter edge-to-edge repair (TEER) of MR remains to be established. Therefore, the key aim of this review is to assess the main applications of SE in patients undergoing TAVI or TEER.

**Methods:**

We searched for relevant studies to be included in the systematic review on PubMed (Medline), Cochrane library, Google Scholar, and Biomed Central databases. The literature search was conducted in February 2022. The inclusion criteria of the studies were: observational and clinical trials or meta-analysis involving patients with AS or MR evaluated with SE (excluding those in which SE was used only for screening of pseudo-severe stenosis) and treated with percutaneous procedures.

**Results:**

Thirteen studies published between 2013 and 2021 were included in the review: five regarding candidates for TEER and eight for TAVI. In TEER candidates, seeing an increase in MR grade, and stroke volume of >40% during SE performed before treatment was, respectively, related to clinical benefits (*p* = 0.008) and an increased quality of life. Moreover, overall, 25% of patients with moderate secondary MR at rest before TEER had the worsening of MR during SE. At the same time, in SE performed after TEER, an increase in mean transvalvular diastolic gradient and in systolic pulmonary pressure is expected, but without sign and symptoms of heart failure. Regarding TAVI, several studies showed that contractile reserve (CR) is not predictive of post-TAVI ejection fraction recovery and mortality in low-flow low-gradient AS either at 30 days or at long-term.

**Conclusion:**

This systematic review shows in TEER candidates, SE has proved useful in the optimization of patient selection and treatment response, while its role in TAVI candidates is less defined. Therefore, larger trials are needed to test and confirm the utility of SE in candidates for percutaneous procedures of valve diseases.

## Introduction

Mitral regurgitation (MR) and aortic stenosis (AS) are the most prevalent valve diseases in Europe (1, 2). The clinical approach toward both valve diseases has changed due to being able to treat them percutaneously in patients with high surgical risk. Transcatheter edge-to-edge repair (TEER) is used for the treatment of either primary or secondary MR treatment (3), while transcatheter aortic valve implantation (TAVI) is used for AS (4). Stress echocardiography (SE) is a valuable tool in the assessment of asymptomatic severe valvular disease used to determine the severity of the disease (5). However, due to lack of robust evidence, its use is not recommended in the guidelines for the management of most valvular diseases (1). In the context of AS, dobutamine SE serves as the cornerstone in confirming a low-flow, low-gradient (LFLG) phenotype (in the case of an ejection fraction below 50%), but it also helps distinguish severe from pseudo-severe AS and evaluate the presence of contractile reserve (CR), defined as an increase of at least 20% in stroke volume (SV) during the examination (1–8). Whereas, in patients with MR, SE is useful to evaluate symptom onset (also in relation to hemodynamical changes and exercise), confirm the severity of MR, assess pulmonary pressure, and evaluate CR. However, historically, SE has been underused in Europe, as shown in the Euro Valve Survey in heart disease (2). In fact, only 3.1% of patients with AS and 2.2–4.6% of patients with MR receive SE in the diagnostic work-up for valve disease diagnosis (2). However, considering the prevalence of frail and severely compromised patients with both MR and AS undergoing transcatheter procedures (6, 7), the use of SE before TAVI or TEER implantation could potentially represent an additional tool leading to a better patient selection. In this context, however, it is important to mention that the presence of concomitant significant MR in patients with LFLG AS worsens the low-flow state, resulting in a reduced forward stroke volume (9). Nonetheless, given its potential positive impact, we performed a systematic review of the literature on the applications of SE in candidates for percutaneous procedures for the treatment of MR and AS. The aim was to highlight the most relevant applications of this diagnostic tool in the contemporary population of patients with heart valve disease who are candidates for TAVI or TEER.

## Methods

### Search strategy and selection criteria

We performed a systematic review of literature following the Preferred Reporting Items for Systematic reviews and Meta-Analyses (PRISMA) statement, updated to 2021 version (10). We included studies regarding the use of SE in (i) candidates for percutaneous procedures for AS (TAVI) or (ii) of MR (TEER). Accordingly, the following terms using Medical subject heading (MeSH) strategy were searched: “(stress echo or contractile reserve or dobutamine) and (TAVI OR TAVR),” “(stress echocardiography or dobutamine echocardiography or exercise echocardiography) and (Mitraclip or (transcatheter mitral valve repair) or (transcatheter edge-to-edge repair) or (TEER) OR (TMVR).” The databases analyzed were PubMed, Biomed Central, Web of Science, Cochrane Library. The literature search was carried out in February 2022. Only full-text article published in English and in peer-reviewed journals were selected. The inclusion criteria of the studies were: observational, clinical trials or meta-analysis involving patients with AS or MR evaluated with SE (excluding those in which SE was used only for screening of pseudo-severe stenosis) and treated with percutaneous procedures. Exclusion criteria: (i) abstract or posters; (ii) reviews or editorials; (iii) rationale and study protocols. The main aim of the systematic review is to summarize relevant studies exploring the role of SE in candidates for TAVI or TEER in defining diagnosis of valvular disease severity and prognosis before or after percutaneous valvular treatment. No limitation in the number of patients included was applied. Literature search, screening of the literature and quality appraisal of selected items was performed by two independent reviewers (GF and NB). Divergences have been solved by discussion and consensus. In case of discordance a third reviewer (RP) was asked to solve the disagreement and reach consensus. The quality of the included studies has been assessed using pre-specified electronic forms of MINORS criteria (11). The minimum score obtained was 16 and the maximum was 20. No studies were excluded based on quality assessment.

## Results

### Search strategy

**FIGURE 1:**
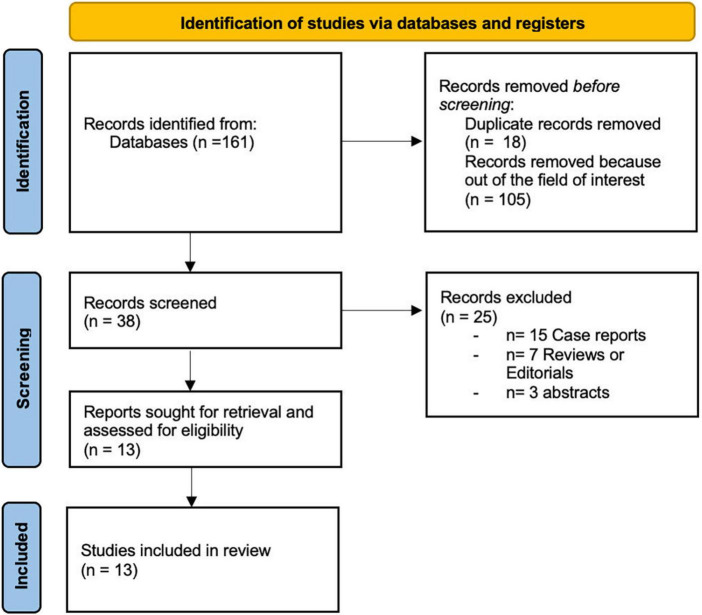
Review flow chart.

Overall, 161 studies were selected (Figure 1).

After a first evaluation, 38 records were screened and of these, 25 were excluded for varied reasons (Figure 1). Finally, 13 studies published between 2013 and 2021 were included in the review (Table 1). In particular five concerned candidates for TEER (349 patients), while eight included candidates for TAVI (895 patients). We presented the results of the systematic review, answering questions related to the use of SE in TAVI and TEER candidates (Tables 1, 2).

**TABLE 1:** Studies on SE in candidates for TEER.

References	Study type	*N*	Role of SE	Type of MR	Stress used	Outcome
Velu et al. (12)	Prospective	39	Assessment of MR grade and LV function during stress before TEER	50% secondary MR	Handgrip or low dose dobutamine	SV increase >40% was associated with better quality of life [improvement on 4/8 subscales of RAND Short Form-36 on Quality of Life was observed: Physical Functioning (*p* < 0.001), Social Functioning (*p* < 0.001), Mental Health (*p* = 0.022) and Vitality (*p* = 0.026)]. Decreased MR grade during SE was associated with worse outcome after TEER (*p* = 0.008).
Curio et al. (13)	Retrospective	221	Verify presence of severe exercise-induced MR	77% secondary MR (75% of severe MR and 84% of moderate MR)	Handgrip	Patients with severe exercise-induced MR may be a group with earlier stage of disease that can benefit from TEER (mortality rate after 2 years of severe exercise-induced MR and severe resting MR were similar with log rank *p* = 0.16). The combined end-point of all-cause death and hospitalization was significantly worst in patients with severe MR at rest (log rank *p* = 0.01). Previous HF hospitalization (log rank *p* < 0.01) and large LVEDd >53.5 mm (AUC 0.696 *p* < 0.001; sensitivity 86% specificity 54%; log rank *p* < 0.001 for cut-off) are prognostic factors.
Izumo et al. (14)	Case-control	46	Verify presence of severe exercise-induced MR	100% secondary MR	Bicycle ergometer	Patients treated with TEER had better event-free survival rate than the ones treated with medical therapy (log rank *p* = 0.017). LVEF during stress (HR 0.919; *p* = 0.028) and TEER procedure (HR 0.419; *p* = 0.044) were independently associated with the composite endpoint of all-cause mortality and hospitalization for HF.
Paranskaya et al. (15)	Prospective	20	Evaluation IMS after multiple TEER	60% secondary 40% primary/mixed	Full-dose dobutamine	During SE, the mean transvalvular gradient increases (3.3 ± 0.8 mmHg vs. 4.0 ± 0.6 mmHg; *p* < 0.001), along with MVOA (2.9 ± 0.3 cm^2^ vs. 3.9 ± 0.4 cm^2^; *p* < 0.001). These results confirm the preserved pliability and elasticity of the valve even after multiple clips implantation.
Boerlage-van Dijk et al. (16)	Retrospective	23	Evaluation presence of IMS after successful TEER	26% degenerative; 74% secondary	Bicycle ergometer	PHT is the only intraprocedural parameter associated with mean transvalvular gradient after TEER with a cut-off of 91 ms (AUC 0.8 *p* < 0.001). During SE, MTDG (from 3.6 ± 1.7 to 6.3 ± 2.7 mmHg, *n* = 23, *p* < 0.001) and sPAP (from 35 ± 12 mmHg to 47 ± 7 mmHg, *n* = 23, *p* = 0.035) increased significantly, but without signs or symptoms of HF or increased BNP levels.

BNP, none natriuretic peptide; HF, heart failure; IMS, iatrogenic mitral stenosis; LV, left ventricular; LVEDd, left ventricular end diastolic diameter; MR, mitral regurgitation; MTDG, mean transvalvular diastolic gradient; MVOA, mitral valve orifice area; PHT, pressure half time; SE, stress echocardiography; sPAP, systolic pulmonary artery pressure; SV, stroke volume; N, number of patients; TEER, transcatheter edge to edge repair.

**TABLE 2:** Studies on SE in candidates for TAVI.

References	Study type	*N*	Role of SE	Stress used	Outcome
Hayek et al. (8)	Retrospective	49	Assessment of contractile reserve	Low-dose dobutamine	30-day mortality is lower in LFLGAS patients with CR prior to TAVI Long term mortality is lower in LFLGAS patients with CR prior to TAVI (HR: 4.47; *p* = 0.037)
Ribeiro et al. (17)	Prospective	234	Assessment of contractile reserve	Low-dose dobutamine	There are no differences in 30-day mortality in LFLGAS patients with or without CR prior to TAVI, although in those with CR mortality tends to be lower (1.2 vs. 5.6% in patients with and without contractile reserve, respectively; *p* = 0.13) There are no differences in long term mortality in LFLGAS patients with or without CR prior to TAVI (28.7 vs. 35.3% in patients with and without contractile reserve, respectively, Log Rank: *p* = 0.704) CR failed to predict LVEF recovery in LFLGAS patients after TAVI Mortality after TAVI in LFLGAS patients is lower than reported in SAVR studies
Buchanan et al. (18)	Retrospective	61	Assessment of contractile reserve	Full dose dobutamine	There are no differences in 30-day mortality in LFLGAS patients with or without CR prior to TAVI (13% in patients with CR vs. 10% in patients without CR; *p* = 1.00) There are no differences in long term mortality in LFLGAS patients with or without CR prior to TAVI (29% in patients with CR vs. 33% in patients without CR; *p* = 0.72) SVi increased significantly after TAVI only in LFLGAS patients with CR prior to TAVI (35% in the group with CR vs. 29% in the group without CR; *p* = 0.04) CR failed to predict LVEF recovery in LFLGAS patients after TAVI (10 vs. 10% in the group with CR and group without CR, respectively, *p* = 0.76)
Sato et al. (19)	Prospective	235	Assessment of contractile reserve	Low-dose dobutamine	LFLGAS with reduced EF, CR or AS severity stratification performed by dobutamine stress echocardiography is not associated with survival nor in patients treated with SAVR nor in those treated with TAVI (HR: 1.09; 95% CI 0.78–1.53; *p* = 0.62)
Maes et al. (20)	Prospective	92	Assessment of contractile reserve	Low-dose dobutamine	There are no differences in 30-days mortality in LFLGAS patients with very low LVEF with or without CR prior to TAVI, although in those with CR mortality tends to be lower (HR 0.47 95% CI 0.04–5.34; *p* = 0.54) There are no differences in long term mortality in LFLGAS patients with very low LVEF with or without CR prior to TAVI (HR 1.37 95% CI 0.58–3.26; *p* = 0.47) CR failed to predict LVEF recovery in LFLGAS patients with very low LVEF after TAVI (mean [SD] relative increase of 27% [35%] vs. 26% [42%]; *p* = 0.95)
Barbash et al. (21)	Prospective	99	Assessment of contractile reserve	Full dose dobutamine	There are no differences in 30-day mortality in LFLGAS patients with or without CR prior to TAVI, although in those with CR mortality tends to be lower (6.25% in patients with CR vs. 30% in patients without CR; *p* = 0.26) There are no differences in long term mortality in LFLGAS patients with or without CR prior to TAVI (25% in patients with CR vs. 40% in patients without CR; *p* = 0.41) CR failed to predict LVEF recovery in LFLGAS patients after TAVI (30% in patients with CR vs. 57% in patients without CR; *p* = 0.35)
Saevik et al. (22)	Prospective	50	Assessment of contractile reserve Safety and feasibility of the exam	Low-dose dobutamine	CR was found in a lower proportion of high-gradient AS patients than in LFLGAS patients’ studies DSE is safe and feasible in high gradient AS patients
D’Andrea et al. (23)	Prospective	75	Assessment of contractile reserve	Low-dose dobutamine	LVGLS values > −12% prior to TAVI are a strong predictor of pre-procedural CR absence (multiple partial correlation coefficient = 0.6, *p* < 0.00001) and lack of post-procedural remodeling in LFLGAS patients (correlation coefficient = 0.44, *p* < 0.0001) Pre-procedural CR predicts a greater increase in LVGLS in LFLGAS patients after TAVI

AS, aortic stenosis; DSE, dobutamine stress echocardiography; CR, contractile reserve; LVEF, left ventricular ejection fraction; LVGLS, left ventricular global longitudinal strain; LFLGAS, low flow-low-gradient aortic stenosis; TAVI, transcatheter aortic valve implantation; SAVR, surgical aortic valve replacement; SVi, indexed stroke volume; SD, standard deviation.

### Stress echocardiography in candidates for transcatheter edge-to-edge repair

Velu et al. (12) showed that patients undergoing SE before TEER (*n* = 36) might have a dual response: in case of MR reduction during SE patients remained in NYHA III–IV or died within 6 months, while 62% (18 out of 29) of the patients with stable or increased MR during SE had clinical benefit (*p* = 0.008), above all in terms of quality of life if an increase in stroke volume during SE in seen (12). Curio et al. (13) instead compared the outcome of 55 patients with moderate MR that become severe during SE with patients with severe MR at rest (*n* = 166), showing that the combined end-point of all-cause death and hospitalization was significantly worst in patients with severe MR at rest. Izumo et al. (14) showed that patients with moderate MR that become severe during SE (*n* = 46) reported a higher event-free survival rate if treated with TEER rather than if medically managed after a 13-month follow-up period (log-rank *p* = 0.017). However, the Cox proportional-hazard analysis suggested that in case of TEER treatment the composite endpoint of all-cause mortality and hospitalization for HF occurred more frequently (hazard ratio: 0.419, *p* = 0.044) (14). Finally, regarding the effect of TEER on mitral valve function, Paranskaya et al. (15), performed dobutamine SE after TEER in 20 patients showing that both mean *trans*-mitral pressure gradient (TPG) (3.3 ± 0.8 mmHg vs. 4.0 ± 0.6 mmHg; *p* < 0.001) and mitral valve orifice area (2.9 ± 0.3 cm^2^ vs. 3.9 ± 0.4 cm^2^; *p* < 0.001) were significantly increased during SE, as well as LVEF (41 ± 18% vs. 46 ± 21%; *p* < 0.001) and systolic pulmonary artery pressure (sPAP) (42 ± 11 mmHg vs. 44 ± 12 mmHg; *p* = 0.014). The degree of MR was stable during stress (*p* = 0.68) (15). Boerlage-van Dijk et al. (16) obtained similar results in terms of increased TPG during SE (from 3.6 ± 1.7 to 6.3 ± 2.7 mmHg, *n* = 23, *P* < 0.001), but also demonstrated that higher TPG and sPAP did not lead to more symptoms of heart failure (16) (Table 1).

### Stress echocardiography in transcatheter aortic valve implantation patients

Hayek et al. (8) showed that in 49 patients with LFLG AS, more than a half (55%) did not have CR and that these patients had worse short- and intermediate-term survival compared with those with CR (log rank *p* = 0.029). Thirty-day mortality was 21 vs. 5%, 1-year mortality 30 vs. 9%, and 2-year mortality 46 vs. 26% compared with those with CR (*p* < 0.001). As opposite, Ribeiro et al. (17) showed that the 45% of 234 patients enrolled in the True or Pseudo-Severe Aortic Stenosis-TAVI registry (TOPAS-TAVI) Registry with LFLG AS had CR, however, the absence of CR at baseline dobutamine SE was not associated with any negative effect on clinical outcomes (30 days, 1 and 2 years mortality) or LVEF changes at follow-up (17). The same results were found by Buchanan et al. (18) (all-cause mortality at 30 days: 13% with CR vs. 10% without CR, *p* = 1.00 and 1 year mortality: 29% with CR vs. 33% without CR, HR 1.20, 95% CI 0.49–2.96, *p* = 0.69) (18) and Sato et al. (19). Maes et al. (20) confirmed that the absence of CR had no effect on clinical outcomes or changes in LVEF over time also in a subgroup of TOPAS-TAVI registry of 92 patients with LVEF < 30% (20). Barbash et al. (21) in 61 patients with LFLG AS undergoing TAVI showed that CR assessed with dobutamine SE did not predict LVEF recovery but did predict lower mortality (21). Saevik et al. (22) demonstrated safety and feasibility of low-dose dobutamine SE in 50 patients with high gradient AS, showing reduced CR in 40% of them (22). Finally D’Andrea et al. (23) showed that a cutoff value for left ventricle global longitudinal strain (LV GLS) of >−12% well distinguished patients without significant CR and with lack of positive remodeling after TAVI at follow-up (23) (Table 2).

## Discussion

### Stress echocardiography in candidates for transcatheter edge-to-edge repair

#### How can stress echocardiography help the clinician select transcatheter edge-to-edge repair candidates?

**FIGURE 2:**
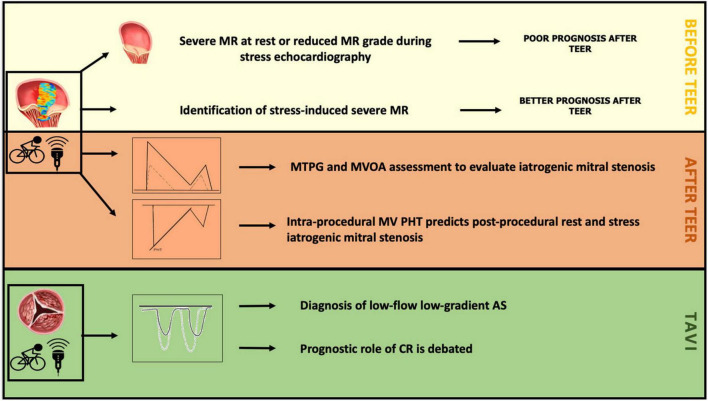
Role of stress echocardiography (SE) in TEER and TAVI. MR, mitral regurgitation; TEER, transcatheter edge-to-edge repair; TAVI, transcatheter aortic valve implantation; MVOA, mitral valve opening area; MTPG, mean *trans*-valvular pressure gradient; MV, mitral valve; PHT, pressure half-time; AS, aortic stenosis; CR, contractile reserve.

To understand the potential usefulness of SE in patients undergoing TEER, it is necessary to consider the lessons learned from the Cardiovascular Outcomes Assessment of the Mitraclip Percutaneous Therapy for Heart Failure Patients with Functional Mitral Regurgitation (COAPT) trial (24), and the Percutaneous Repair with the Mitraclip Device for Severe Functional/Secondary Mitral Regurgitation (MITRA-FR) trial (25), both contributing to the elaboration of the concept of proportionate and disproportionate MR (26). This concept helped clinicians distinguish to what extent symptoms are caused by the amount of MR or by left ventricular dysfunction (26). In this context, SE might be useful to evaluate (i) the changes of the left ventricle functional reserve during stress; (ii) the variation of MR grade during exercise; (iii) the changes in pulmonary pressures; (iv) the onset of symptoms in relation to hemodynamical changes. These measurements can change the decisional process that leads to an indication for TEER (Figure 2).

In a small prospective study (*n* = 36), Velu et al. (12) enrolled patients who underwent SE with handgrip and/or dobutamine before successful TEER with Mitraclip (50% secondary MR). The aim of the study was to evaluate which parameters were associated with mortality or NYHA class >II in a 6-month follow-up. The endpoint occurred in 18 patients (50%). The seven patients with decreased MR grade during SE remained in NYHA III-IV or died within 6 months, while 62% of patients with stable or increased MR grade during SE reported clinical benefits (*p* = 0.008) (12). A left ventricular stroke volume increase of >40% during stress was associated with an increased quality of life after the procedure, and patients who did not achieve this goal had a higher heart rate during stress and rest (12). The study shows that if MR is maintained during SE, the patient’s outcome is better. In fact, the absence of an increase in stroke volume during SE is related to a critical dysfunction of the left ventricle, which cannot adapt stroke volume to stress, resulting in an increase in heart rate to compensate for the lack of positive inotropism (12). At the same time, a decreased grade of MR during SE is indicative of the fact that MR is a minor determinant of the patient symptoms during exercise compared to the abnormal left ventricle function (12). The study of Velu et al. (12) also showed that the endpoint occurred in a high number of patients (50%), reflecting the severity of the underlying disease.

Curio et al. (13) tried to understand if SE might be useful in selecting patients at an early stage of the disease, thus truly benefiting from TEER (77% of patients with secondary MR). This retrospective study (13) (*n* = 221) explored the effect of TEER treatment in patients with handgrip-induced severe MR and patients with severe MR at rest. Patients with handgrip-induced severe MR were considered as having an earlier stage of the disease. The primary endpoint was all-cause mortality and HF hospitalization in a follow-up of 2 years (13). Patients with severe MR at rest had a significantly lower left ventricular ejection fraction (LVEF), larger left ventricle end-diastolic diameter (LVEDd) and larger left atrial volumes compared to patients with severe handgrip-induced MR (13). However, MR improvement after TEER was greater in patients with severe resting MR, even if it was significant in both groups, resulting in a reduction of systolic pulmonary artery pressure (sPAP) after procedure (13). The 2-year mortality rate was 4% in patients with handgrip-induced severe MR and 8% in patients with severe resting MR (13). Patients with severe resting MR suffered more frequently from the combined endpoint (*p* = 0.01), and the difference was driven by HF hospitalization (*p* = 0.02). However, in both groups, a significant reduction of HF hospitalizations in the 2 years after the procedure compared with the 2 years before the procedure was achieved (13). Independent predictors of worse outcomes were preprocedural hospitalization for HF (*p* < 0.01) and larger LVEDd with a cutoff of 53 mm (AUC 0.696, sensitivity 86%, specificity 54%) (13). Patients with residual MR greater than mild showed a worse 2-year outcome (13). These results suggest that in patients at an early stage of MR disease, the outcome after the TEER procedure is better (13). However, it does not explain whether MR severity itself or LV remodeling caused by MR are responsible for the prognostic divergence between groups.

In another study, Izumo et al. (14) enrolled patients (*n* = 46) with exercise-induced MR (defined as an increase in effective regurgitant orifice area (EROA) ≥0.13 cm^2^ during semi-supine bicycle ergometer), which have been then treated with TEER or with conservative management (14). All patients enrolled had secondary MR. At baseline, patients had an EROA of 0.26 ± 0.10 cm^2^ and 0.20 ± 0.08 cm^2^, and a regurgitant volume of 42 ± 13 ml and 33.6 ± 13.6 ml in the TEER group compared to those treated with conservative management, respectively. The authors specified that the decision to treat with TEER or conservatively was left to the clinician and related to routine clinical practice. The primary endpoint was the occurrence of death and hospitalization (14). The mean follow-up was 13 months. Patients treated with TEER were older, with a higher prevalence of NYHA ≥ II, smaller left ventricle and higher LVEF compared to those in the optimal medical therapy (control) group (14). However, there were no differences in stroke volume and cardiac output between the groups. Event-free survival was higher in the TEER group (Log Rank *p* = 0.017), reaching 82% at 12 months and 56% at 24 months (14). LVEF during exercise (HR 0.919, *p* = 0.028) and TEER (HR 0.419, *p* = 0.044) were independently associated with clinical outcomes. Moreover, 25% of patients with moderate secondary MR at rest showed worsening of MR during exercise. The event-free survival of these patients was similar to those with severe MR at rest (14). These patients, who might benefit from TEER, are underestimated in clinical practice due to the underuse of SE.

In conclusion, the benefits of using SE are twofold. First, SE may help predict the outcome of patients after TEER treatment. Second, it helps identify patients with an earlier stage of the disease who may undergo TEER with a greater benefit.

#### How can stress echocardiography evaluate anatomical response to transcatheter edge-to-edge repair?

Transcatheter edge-to-edge repair procedure is guided by transesophageal echocardiography (TOE). TOE is necessary to choose the correct position during clip releasing and check for the presence of residual MR or excessive increase in *trans*-valvular diastolic gradient in order to avoid iatrogenic mitral stenosis (IMS) (defined by a post-TEER diastolic gradient ≥5 mmHg) (Figure 2) (16). The reliability of the measures of MR or IMS done during the TEER is debatable because influenced by the general anesthesia and loading conditions. Doppler measurements are operator dependent and strongly influenced by left ventricle function, left atrial compliance, and loading conditions, and real-time monitoring evaluation of left atrial pressure during TEER is proven to be able to predict the outcome independently from echocardiographic findings (27). Therefore, considering this, although SE in clinical practice is not often used in patient evaluation after TEER some authors considered it to better explain echocardiographic findings after the procedure. Boerlage-van Dijk et al. (16), in a retrospective study (*n* = 51, but only 23 underwent SE, of whom 74% with secondary MR), investigated which echocardiographic parameters were associated with iatrogenic mitral stenosis (IMS) during TEER implantation with TOE and after with SE in 13-month follow-up. During intra-procedural assessment mean transvalvular diastolic gradient (MTDG) was higher after the procedure than during the procedure (*p* < 0.001), while pressure half-time (PHT) did not change significantly (16). PHT was the only intraprocedural parameter that predicted post-procedural IMS with good accuracy (AUC 0.9) using a cut-off of 91 ms (16). However, during SE performed after TEER with bicycle echocardiography, MTDG and sPAP increased significantly, but without signs or symptoms of HF or any significant difference in brain natriuretic peptide (BNP) plasma level (16). The change of these parameters after TEER was comparable to that described after surgical edge-to-edge mitral valve repair technique (16).

Finally, Paranskaya et al. (15) performed a small prospective study to evaluate the impact of multiple clips on mitral valve function and *trans*-mitral gradients. They included in the study 20 patients (60% with secondary mitral regurgitation) with residual less than moderate MR, but with increased MTDG [from 2.3 ± 0.1 (range 1.0–4.5) to 3.3 ± 0.8 (range 1.8–5.0) mmHg; *p* = 0.002] and reduced mitral valve orifice area (MVOA) [from 5.8 ± 0.9 (range 4.0–7.6) to 2.9 ± 0.3 (range 2.5–3.6) cm^2^; *p* < 0.001] after TEER. Under SE, both MTDG and MVOA significantly increased, supporting the hypothesis of a preserved pliability and elasticity of the valve after multiple clip implantation (15).

### Stress echocardiography in transcatheter aortic valve implantation patients

#### How can stress echocardiography help the clinician select patients with low-flow, low-gradient aortic stenosis who are candidates for transcatheter aortic valve implantation?

As stated before, SE is the gold standard for CR assessment in patients with LFLG-AS with EF < 50% (1–8). However, the prognostic role of CR for patients who are candidates for TAVI or SAVR has been questioned due to recent data. In LFLG-AS patients undergoing SAVR, the peri-procedural risk was considered greater if CR was absent (17). Data from the Transcatheter Aortic Valve Replacement in Patients With Low-Flow, Low-Gradient Aortic Stenosis (TOPAS-TAVI) registry published by Ribeiro et al. (17) as well as the findings from the sub-analysis of Maes et al. (20) and other smaller studies (18, 21) do not confirm these results for patients undergoing TAVI, although in patients with CR there was a trend toward lower 30-day mortality (1.2 vs. 5.6% in patients with and without CR, respectively; *p* = 0.13). Reasons for that might be related to the less invasive nature of the procedure, requiring less postoperative care, and being the percutaneous procedure altogether better tolerated by the patient (18, 20). Contrastingly, Hayek et al. (8) showed that 30-day mortality was significantly lower in patients with CR treated with TAVI compared to patients without CR (5 vs. 21%, *p* < 0.001). It should also be remembered that the population of Hayek et al. (8) consisted of a small and very heterogeneous sample, also including patients with preserved left ventricular ejection fraction LVEF (23% of the sample population), while the other studies enrolled only patients with reduced LVEF (17, 18, 20, 21). Finally, Sato et al. (19) showed that in LF-LG AS with reduced ejection fraction, CR or AS severity stratification performed by dobutamine stress echocardiography was not associated with survival, neither in patients treated with SAVR nor in those treated with TAVI (19).

In conclusion, data reveal a discordancy about the efficacy of CR in predicting the outcome in LFLG AS for both TAVI and SAVR candidates. As seen above, mortality in patients undergoing TAVI is not related to CR presence, either at 30 days (although there is a tendency for a better peri-procedural outcome in patients with CR) or at long term (17, 18, 20, 21). However, the total mortality remains lower for TAVI patients without CR compared to that reported in SAVR studies (17). Furthermore, since CR is not predictive of the outcome of TAVI patients, TAVI should not be discouraged by an absence of pre-procedural CR (17) (Figure 2). Finally, no studies specifically address how to use SE in the evaluation of patients undergoing TAVI with LFLG AS and concomitant severe MR, or specifically with LFLG AS with LVEF higher than 50% [besides the few patients enrolled in Hayek et al. (8) study].

#### How can stress echocardiography help the clinician select patients with high-gradient aortic stenosis who are candidates for transcatheter aortic valve implantation?

In classic high-gradient AS, SE is not usually performed and there is little data about it. Saevik et al. (22) published an initial analysis of a cohort of patients with symptomatic high-gradient AS and LVEF > 40% in which pre-procedural CR was evaluated with a low-dose DSE pre-TAVI. The aim was to evaluate the safety and feasibility of the DSE in this subset of patients and look for the presence of CR (22). Of the 50 patients enrolled, 45 (90%) completed the protocol. Only 10% of them reported minor events that caused test interruption (22). Symptoms rapidly regressed with dobutamine suspension (22).

Ten (20%) patients showed low indexed stroke volume (SVi) before the exam (22). CR was found in 20 (40%), with an average increase in SVi of 32%. Interestingly, no difference in the prevalence of CR between patients with low-flow and normal-flow at baseline was found nor was a relationship with NT-proBNP or diastolic parameters or mass (22). The proportion of patients with CR was lower in high-gradient AS patients than in LFLG patients, but this could be explained by a higher SVi (43 ± 10 ml/m^2^) at baseline and by the presence of preserved LVEF (baseline LVEF 66%) (22).

#### Can contractile reserve predict long-term outcome in low-flow, low-gradient patients undergoing transcatheter aortic valve implantation?

Several studies tried to correlate the long-term outcome of LFLG patients undergoing TAVI with the presence of CR at pre-procedure dobutamine SE (DSE), but the results are conflicting (17, 18, 20, 21).

Barbash et al. (21) showed how the advantage in terms of reduced mortality changes over time in patients with CR, progressively decreasing until reaching an overlap of the two mortality curves in patients with and without CR. Similarly, both in the TOPAS-TAVI registry (with a mean follow-up of 21 months), and in the Buchanan et al. study (follow-up = 12 months), patients with LVEF < 30% showed the absence of a significative correlation between the presence of pre-procedural CR and long-term mortality (17, 18, 20, 21).

Discordant results come from the Hayek et al. (8) study, where 81% of the dead patients did not show CR at the pre-procedure DSE (1-year mortality 30 vs. 9% and 2-year mortality 46 vs. 26% of the CR group vs. no-CR group, respectively, *p* < 0.001) (8). As pointed out by Ribeiro et al. (17), however, a large part of long-term deaths in patients undergoing TAVI are non-cardiac, and mainly due to comorbidities, such as chronic obstructive pulmonary disease (COPD), or pre-operative anemia (mainly driven by iron deficiency). Considering this, it is important to note that in the Hayek et al. (8) study the only significant difference between the two groups stratified by the presence of CR at the baseline was precisely the higher prevalence of COPD in patients without CR (63 vs. 32%; *p* = 0.030). This may be enough to explain the higher mortality, despite the multivariate analysis showing both COPD and CR as independent predictors of mortality (8, 17). After TAVI, Anjan et al. (28) demonstrated that an increase in SVi is related to an improvement in 1-year survival. Buchanan et al. (18) showed that SVi significantly increases after TAVI only in patients with CR. In this setting, DSE demonstrated low (≤65%) specificity and sensitivity in predicting it (28). Finally, left ventricular global longitudinal strain (LVGLS) was analyzed as a parameter useful to predict the presence of CR and the post-TAVI reverse remodeling (23), showing that a pre-procedural LVGLS > −12% was a strong predictor of pre-procedural CR absence (sensitivity 84%; specificity 93%; AUC 0.92 [95% CI, 0.86–0.99], *p* < 0.00001) and lack of post-procedural remodeling (*p* < 0.0001) (23). A greater increase in the absolute value of LVGLS after TAVI has been highlighted in patients with pre-procedure CR (+3.9%, *p* < 0.0001 vs. + 2.3%, *p* = 0.01) (23).

#### Clinical perspectives and limitations of systematic review

**FIGURE 3:**
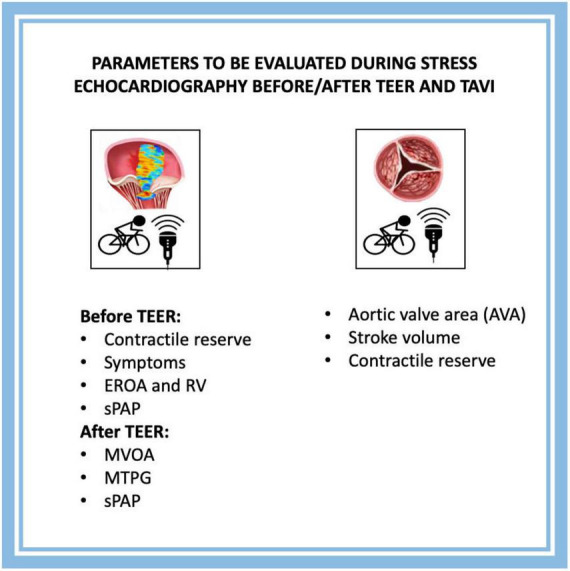
Parameters to be evaluated during SE before/after TEER and TAVI. TEER, transcatheter edge-to-edge repair; TAVI, transcatheter aortic valve implantation; EROA, effective regurgitant orifice area; RV, regurgitant volume; sPAP, systolic pulmonary arterial pressure; MVOA, mitral valve opening area; MTPG, mean *trans*-valvular pressure gradient.

The main limitations of studies about the use of SE in candidates for TAVI or TEER are related to the small size of the samples, the absence of randomized design, and not standardized SE protocols. Considering all of this, a formal meta-analysis of data was not possible. When using SE in candidates for TEER we expect (Figure 3) the confirmation of indication through the evaluation of symptoms during stress, and the quantification of worsening of MR or elevated sPAP. We also expect to test the possible positive effect of TEER mainly with CR assessment. After TEER in case of IMS, SE is of paramount importance to understand if the increased MTPG is or not related to HF.

Regarding candidates for TAVI, the only clear indication to SE is in the context of LFLG AS to confirm a diagnosis of stenosis (Figure 3). The role of CR is still uncertain and not necessary for an indication to TAVI. New trials are needed to understand if SE might be relevant also for patients with paradoxical low-flow, low-gradient AS or with combined valvular disease (e.g., MR and AS).

## Conclusion

This systematic review shows that there are few studies analyzing the role of SE in candidates for TAVI and TEER. In a TEER context, SE is useful before TEER and in patients with secondary mitral regurgitation for two reasons. First, to identify patients developing severe MR during SE who are in an early phase of the disease and might benefit from an earlier intervention. Second, to identify patients with reduced CR and MR who have more advanced disease and poor prognosis. Conversely, in TAVI candidates, the role of SE is limited to confirming the severity of the diagnosis in the case of LFLG AS and reduced LVEF (as established by recommendations). New studies are needed to better explore the role of SE after TEER and verify if SE might be relevant also for patients with paradoxical low-flow, low-gradient AS, or with combined valvular disease.

## Data availability statement

The original contributions presented in this study are included in the article/supplementary material, further inquiries can be directed to the corresponding author.

## Author contributions

RP, ET, GP, and GC: conception, design, analysis, and interpretation of data. GF, NB, FS, MD, LZ, RP, GC, and MS: drafting of the manuscript and revising it critically for important intellectual content. MS, GC, and RP: drafting of the manuscript and revising it critically for important intellectual content, data collection, and verification of data. All authors contributed to the final approval of the manuscript submitted.
